# The influence of micrometastases on prognosis and survival in stage I-II colon cancer patients: the Enroute*⊕ *Study

**DOI:** 10.1186/1471-2482-11-11

**Published:** 2011-05-11

**Authors:** Daniel J Lips, Boukje Koebrugge, Gerrit Jan Liefers, Johannes C van de Linden, Vincent THBM Smit, Hans FM Pruijt, Hein Putter, Cornelis JH van de Velde, Koop Bosscha

**Affiliations:** 1 Department of Surgery, Jeroen Bosch Hospital, Nieuwstraat 34, 5211 NL 's-Hertogenbosch, the Netherlands; 2 Department of Surgery, Leiden University Medical Center, PO Box 9600, 2300 RC Leiden, the Netherlands; 3 Department of Pathology, Jeroen Bosch Hospital, Nieuwstraat 34, 5211 NL 's-Hertogenbosch, the Netherlands; 4 Department of Pathology, Leiden University Medical Center, PO Box 9600, 2300 RC Leiden, the Netherlands; 5 Department of Medical Oncology, Jeroen Bosch Hospital, Nieuwstraat 34, 5211 NL 's-Hertogenbosch, the Netherlands; 6 Department of Medical Statistics, Leiden University Medical Center, PO Box 9600, 2300 RC Leiden, the Netherlands

## Abstract

**Background:**

The presence of lymph node metastases remains the most reliable prognostic predictor and the gold indicator for adjuvant treatment in colon cancer (CC). In spite of a potentially curative resection, 20 to 30% of CC patients testing negative for lymph node metastases (i.e. pN0) will subsequently develop locoregional and/or systemic metastases within 5 years. The presence of occult nodal isolated tumor cells (ITCs) and/or micrometastases (MMs) at the time of resection predisposes CC patients to high risk for disease recurrence. These pN0_micro+ _patients harbouring occult micrometastases may benefit from adjuvant treatment. The purpose of the present study is to delineate the subset of pN0 patients with micrometastases (pN0_micro+_) and evaluate the benefits from adjuvant chemotherapy in pN0_micro+ _CC patients.

**Methods/design:**

EnRoute+ is an open label, multicenter, randomized controlled clinical trial. All CC patients (age above 18 years) without synchronous locoregional lymph node and/or systemic metastases (clinical stage I-II disease) and operated upon with curative intent are eligible for inclusion. All resected specimens of patients are subject to an *ex vivo *sentinel lymph node mapping procedure (SLNM) following curative resection. The investigation for micrometastases in pN0 patients is done by extended serial sectioning and immunohistochemistry for pan-cytokeratin in sentinel lymph nodes which are tumour negative upon standard pathological examination. Patients with ITC/MM-positive sentinel lymph nodes (pN0_micro+_) are randomized for adjuvant chemotherapy following the CAPOX treatment scheme or observation. The primary endpoint is 3-year disease free survival (DFS).

**Discussion:**

The EnRoute+ study is designed to improve prognosis in high-risk stage I/II pN0 _micro+ _CC patients by reducing disease recurrence by adjuvant chemotherapy.

**Trial Registration:**

ClinicalTrials.gov: NCT01097265

## Background

Colorectal cancer is the second most commonly diagnosed malignancy in men and women in the Netherlands with increasing incidence due to growth and ageing of the general population [1]. The presence of lymph node metastases remains the most reliable prognostic predictor and the gold indicator for adjuvant treatment in colon cancer (CC) [2]. Interestingly, in a large percentage of patients without lymph node metastases in the surgical specimen, who are therefore not subjected to adjuvant chemotherapy, represent with disease recurrence. In about 10% of the patients with stage I (Dukes A) and 15-30% with stage II (Dukes B) disease recurrent locoregional or distant metastases develop within 5 years [2-4]. One possible factor could be the presence of occult lymph node metastases at the time of presentation and surgical resection. Evidence has emerged showing a significant amount of nodal metastases being smaller than 2 mm or less (<0.2 mm isolated tumor cells [ITC]; 0.2 - 2 mm micrometastasis [MM]) and, therefore, likely to be missed during conventional gross pathological specimen examination. }[3-5] Focused examination methods, such as more extensive nodal examination by serial sectioning or step sectioning, or molecular detection of metastatic nodal cells by immunohistochemistry (IHC) or reverse transcriptase-polymerase chain reaction (RT-PCR), increase the likelihood of finding these tumour deposits. However, these focused examination methods are expensive and time consuming, and therefore not applicable to all lymph nodes derived from the surgical specimen. By using sentinel lymph node mapping (SLNM) the nodes at highest risk of harbouring tumour deposits can be potentially detected and more thoroughly examined. The *ex vivo *SLNM procedure is technically easy, as the procedure is executed extracorporally by injection peritumoral blue dye subserosally or submucosally after which gentle massage of the injection site is performed [5-11]. Blue coloured lymph nodes are excised or marked by sutures. The *ex vivo *SLNM procedure is characterized by a high accuracy of 90-100%, and negative predictive value of 80-100%[6-11]. A rate of 19-57% upstaging is observed [6-11].

Current knowledge about the prognostic relevance of nodal micrometastases and isolated tumor cells has adequately been reviewed in 2004 separately by Iddings and Nicastri [3,4]. They concluded that a suggestion of prognostic relevance could be made for micrometastatic disease related to worsened disease-free survival (DFS) and overall survival (OS). The meta-analysis performed by Iddings showed a decreased 3-year DFS and OS of respectively 78% and 78% in pN0_micro+ _patients compared to 90% and 97% in pN0_micro- _patients [4]. New evidence from two large international prospective observational studies shows a clear negative prognostic effect of micrometastatic nodal disease [5]. The 4-year DFS detoriated from 94% to 78% with the presence of nodal micrometastasis [5]. A solid conclusion, however, could not be made because of the lack of well-designed, well powered, controlled clinical trials. Data from other solid organ malignancies like breast cancer links micrometastatic disease to a worsened prognosis [12]. Several prospective trials are currently recruiting (see table 1). However, an randomized, controlled clinical trial of significant magnitude is needed to answer this clinically relevant question.

**Table 1:** Currently recruiting studies

University Hospital, Strasbourg, France (C. Brigand)	2007 - 2014	N = 140
Jonsson Comprehensive Cancer Center (A. Bilchik)	2009 - 2011	N = 300
John Wayne Cancer Institute (S. Baker)	2000 - 2011	N = 200
University Hospital, Basel, Switzerland (C. Viehl)	2004 - 2007	N = 225

Source: http://www.clinicaltrials.gov

Nowadays, adjuvant chemotherapy is only offered to high risk stage I-II colon cancer patients in the Netherlands [2]. Stage I-II CC patients without risk factors are thought not to benefit from adjuvant treatment. Individual randomized trials did not show a significant survival benefit in stage II colon cancer patients [2]. The results of meta-analyses and systematic reviews show at the most a slight disease-free survival benefit of adjuvant chemotherapy in stage II disease [2]. However, to stress out the importance of further investigation, stage I-II CC patients do suffer from disease recurrence and their overall 5-year survival is just around 70-80%. Even stage II CC patients without risk factors have been shown in several studies to benefit from adjuvant treatment [13,14]. It is because of these results that in Eastern countries and the United States stage II CC patients with or without micrometastatic disease do receive adjuvant treatment. Thus, there is an international need for better delineation of high-risk stage I-II CC patients who probably should be offered adjuvant treatment.

## Methods/Design

### Study objectives

To determine the benefits from adjuvant chemotherapy in pN0_micro+ _CC patients on 3-year disease-free survival compared to non-treated pN0_micro+ _CC patients.

### Primary endpoint

Primary endpoint is 3-year DFS in study groups (proportion of patients without local or distant recurrence, or second primary of same of other cancer, death from same cancer of any other cause during the defined period of time).

### Secondary endpoints

Secondary endpoints are rate of upstaging in pN0 colonic cancer patients (total number pN0_micro+ _patients × 100/total number pN0 colon cancer patients) and 3-year overall survival.

### Design

**Figure 1:**
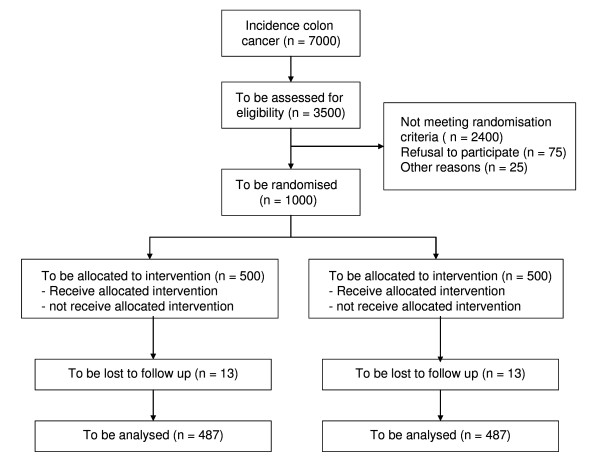
EnRoute+ study according to CONSORT

**Figure 2:**
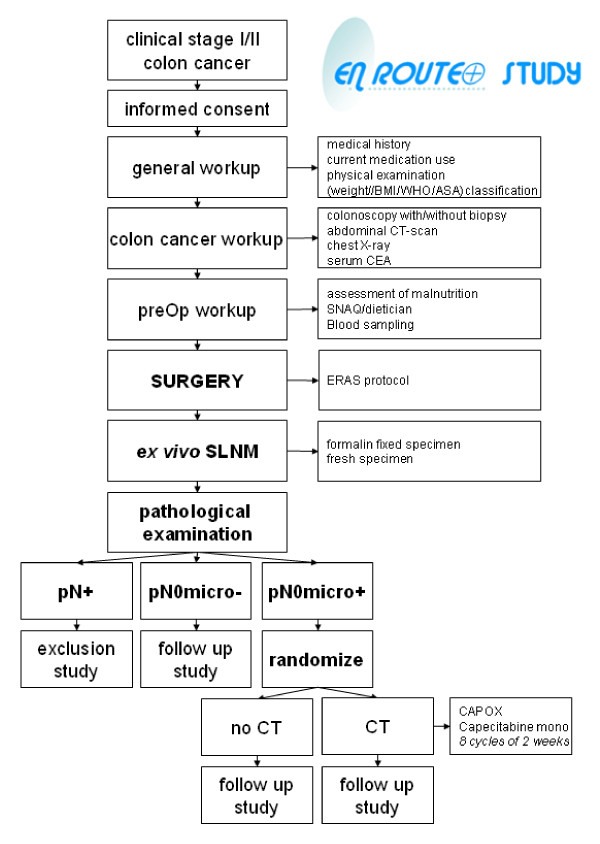
EnRoute+ flow chart

**Figure 3:**
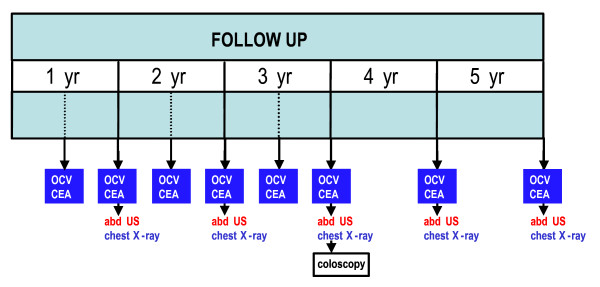
**EnRoute+ follow up**. OCV: outpatient clinic visit. CEA: carcinoma embryonic antigen abd. US: abdominal ultrasonography.

EnRoute+ is an open label, multicenter, randomized controlled clinical trial. A centrally-performed, computer-generated randomization procedure is instituted. Eligible patients are randomly assigned at a 1:1 ratio to receive treatment respectively without or with chemotherapy using block-randomisation per participating center. Design, flow chart and follow up are presented in Figures 1, 2 and 3.

Study groups are defined according the randomisation group and pathological examination result.

group A   pN0_micro+ _with chemotherapy

group B   pN0_micro+ _without chemotherapy

group C   pN0_micro- _without chemotherapy

### Setting

Subjects will be enrolled in multiple academic and non-academic (teaching and non-teaching) hospitals in the Netherlands.

### Patients

A total of 1000 adult patients of both sexes with pN0_micro+ _will be randomized to receive adjuvant chemotherapy.

### Eligibility criteria

#### Inclusion criteria for randomization

- pN0_micro+ _colon cancer patients (stage I/II, Dukes A/B)

- patients deemed to be fit for chemotherapy treatment (WHO classification ≤ 1; ASA classification ≤ 2)

#### Exclusion criteria for randomization

- rectal cancer

- previous chemotherapy

- high risk pN0 patient according to:

○ less then 10 lymph nodes detected in resected specimen, or

○ invasion in other organs (T4NxMx), or

○ colon perforation at presentation, or

○ clinically relevant obstruction at presentation, or

○ angioinvasion at conventional pathological examination

- positive nodal involvement or advanced disease (stage III/Dukes C/Dukes D)

- haematological impairments (anemia, leucopenia, thrombocytopenia within 7 days before start of chemotherapy treatment)

○ renal function impairments (creat.clearance ≤ 60 ml/min within 7 days before start of chemotherapy treatment)

- liver enzyme serum alterations within 7 days before start of treatment

- other current serious illness or medical conditions

○ severe cardiac illness (NYHA class III-IV, see Appendix)

○ significant neurologic or psychiatric disorders

○ uncontrolled infections

○ active disseminated intravascular coagulation

○ other serious underlying medical conditions that could impair the ability of the patient to participate in the study

- definite contraindications for the use of corticosteroids

- use of immunosuppressive or antiviral drugs

- any other experimental drugs within a 4-week period prior to start of surgery and adjuvant chemotherapy and/or throughout/during the study period

- pregnancy or lactation

- patients unable to comply with requirements of the study

### Ethics, informed consent

This study is conducted in accordance with the principles of the Declaration of Helsinki and 'good clinical practice' guidelines. The independent central ethics committee approved the final protocol on 26^th ^July 2010. Oral and written informed consent is obtained from the patient before inclusion in the trial.

### Safety

No additional risks for patients are introduced by the *ex vivo *SLNM procedure as the complete procedure is performed extracorporally. Participation in the randomization study brings risks and discomfort for the patient common to adjuvant systemic chemotherapeutic treatment as given to patients with stage III colon cancer (CAPOX scheme). Dose limiting toxicities of oxaliplatin/capecitabine include diarrhoea, abdominal pain, nausea, stomatitis and hand-foot syndrome (hand-foot skin reaction, palmar-plantar erythrodysesthesia). Most adverse reactions are reversible and do not require permanent discontinuation of therapy, although doses may need to be withheld or reduced. A potential negative effect on prognosis can be expected in patients not receiving adjuvant chemotherapy.

### Statistical analysis

#### Intention-to-treat

The analysis will be performed on the basis of an intention-to-treat (ITT) population and with respect to ITT principles. Also a per-protocol (chemotherapy versus no adjuvant treatment) analysis will be performed.

#### Interim analysis

An interim analysis will be performed when half of the required number of events have been observed. Based on the O'Brien-Fleming alpha-spending function, the first analysis will be performed at nominal alpha level 0.003, the second at nominal alpha level 0.047, to ensure an overall alpha of 0.05.

#### Sample size

The sample size is calculate to be (N=) 973 patients (control group [B] 486 pts/145 events, treatment group [C] 487 pts/102 events with 90% power and a significance level of 0.05. Loss to follow up is expected to be 26 patients. Median follow up for primary outcome is 3 years. Accrual rate is expected to be at least 300 patients/year, which results in a maximum total accrual time of 4 years. Median follow up of 3 years is reached after 5 years total study time.

#### Treatment program

Eligible patients are selected and informed by local clinicians. Written consent is obtained and the patient is prepared for surgery according to the ERAS protocol. The method of surgery (i.e. laparoscopic or open) is chosen by the surgeon together with the patient. The datacenter is informed by the treating clinician about registration of the patient into the trial and the preoperative CRF is send to the datacenter. The operation is performed by a certified surgeon, or supervised surgical resident. Surgery is performed according to anatomical and oncological principles dictated by the location of the tumour. The adjacent mesocolon is dissected including its base and excised en-block. Within fifteen minutes of the resection the *ex vivo *SLNM is performed. The specimen is opened at the anti-mesenteric side. When the tumour is identified, 1 ml of Patent Blue V dye is injected into the submucosa circumferentially to the tumour, using a tuberculin syringe. The injection sites are then gently massaged for up to five minutes to push the tracer into the lymphatic vessels. Blue coloured sentinel nodes are identified and marked with a suture. The specimen is transferred fresh to the pathology laboratory, when possible according to local pathological facilities, for further examination and processing. The specimen is put in formalin within 15 minutes after the SLNM procedure, when local pathological facilities dictate formalin embedded transportation of the specimen. If fresh received, a sample is taken from the primary tumor for tumor banking and side-study purposes. The pathologist examines the specimen using conventional methods and identifies the blue stained nodes and puts them into marked cassettes so that they can be examined by microscopic examination. Sentinel and non-sentinel lymph nodes are bivalved. Each section is analyzed using conventional histologic staining with hematoxylin and eosin (H&E). Efforts are made to analyze at least 10 lymph nodes per specimen. Detection of lymph node metastases is performed by microscopic examination in sentinel and non-sentinel nodes. The patient is excluded for focussed examination, when macroscopic metastases are present (pN+) or angioinvasion is detected (see exclusion criteria). In pN0 patients sentinel lymph nodes are additionally sliced at four levels at 150 μm intervals. All sections are stained with Pan-Cytokeratin (LU-5). Positive cytokeratin lymph node metastases are defined as ITC <0.2 mm or MM 0.2 - 2 mm (see Table 2). Rare single cells staining positive with IHC that lack cytologic characteristics of malignancy are considered tumor-negative. Tumours are staged according to the AJCC TNM classification 2002 (Sixth Edition). Adjuvant chemotherapy is provided according to the CAPOX (XELOX) protocol (capecitabine (Xeloda^®^) and oxaliplatin(Eloxatin^®^)).

**Table 2:** Definitions of isolated tumor cells (ITCs) and micrometastases (MMS)

Definitions used in EnRoute*⊕ *Study		
	Isolated Tumor Cells (ITCs)	Micrometastases (MMs)
Size	< 0.2 mm	0.2 - 2 mm
Localization	No defining criterion	No defining criterion
Detection method	HES and/or IHC	HES and/or IHC
Formal designation (present vs absent)	pN0_i+ _vs pN0_i-_	pN0_mi+ _vs pN0_mi-_
Designation for study purposes if present	pN0_micro+_	pN0_micro+_

HES hematoxylin and eosin stain; IHC immunohistochemistry.

### Monitoring

Research nurses monitor the participating centres and patients. Every 6 months a random selection of participating centres is visited by research nurses checking, at least, 10% of patient's data.

### Follow-up

Patients are followed during their hospital stay. Patients are kept in follow up for five years according to national guidelines (see Figure 3) [2].

## Discussion

Recurrent locoregional and/or systemic disease in stage I/II colon cancer is a significant clinical and social health care problem. The fact of high disease recurrence in stage I-II colon cancer patients is the basis of the study idea and the justification for the study. Around 7000 new colon cancer patients are diagnosed yearly in the Netherlands at this moment and the incidence will rise in the coming decades [2]. Of these new colon cancer patients respectively 16 and 38% have stage I or stage II disease [15]. Therefore, yearly approximately 3500 new stage I-II patients are at risk for disease recurrence. A upstaging rate of 30% is expected to be observed by using SLNM, serial sectioning and IHC for micrometastatic disease detection [6-11]. Approximately 1050 are at high risk for disease recurrence. Nowadays, adjuvant chemotherapy is only offered to high risk stage II colon cancer patients in the Netherlands [2]. Stage II CC patients without risk factors are thought not to benefit from adjuvant treatment. Individual randomized trials did not show a significant overall survival benefit in stage II colon cancer patients [2]. The results of meta-analyses and systematic reviews show at the most a slight disease-free survival benefit of adjuvant chemotherapy in stage II disease of 3-6% absolute risk reduction in disease-free survival (3.8 - 6% ARR in 5-yr DFS by Figueredo et al, 3% ARR in 5-yr DFS in the IMPACT-B2 study, 4% ARR in 5-yr DFS in the Gill-study [2,16-18].

According to SEER data 5-year overall survival data of stage IIa (T3N0M0), IIb (T4N0M0) and IIIa (T1-2N1M0) and b (T3-4N1M0) are respectively 84.7%, 72.2%, 83.4% and 64.1% [19]. The MOSAIC-trial observed a 4-year DFS of untreated versus treated colon cancer stage II and III of respectively 85-87.6% and 66.5-74.1% [20]. The XELOXA-trial found a 3-year DFS of 66.5-70.9% (untreated versus treated) in stage III colon cancer [21]. An 9% absolute risk reduction on 5-year OS (85.4% in the surgery-only group and 94.2% adjuvant chemotherapy group) was observed in T3N0M0 patients following adjuvant chemotherapy in a large retrospective study [13]. A similar result was observed in 468 stage II patients (i.e. 8% 4-year OS risk reduction of 70% to 78%) [22]. In an Australian population-based cohort-study patients with good prognosis (T3, no lymphovascular invasion) showed improved 5-year survival rate with chemotherapy from 85 to 95% (P = 0.064, log-rank test) in women but showed no change in men (84 vs 82%). The poor prognosis (T4 and/or presence of lymphovascular invasion) patients, survival rates for both women (65 vs 79%) and men (72 vs 78%) improved with the use of chemotherapy, however, did not reach significance in either group (P = 0.22 and P = 0.26, respectively) [14]. It is therefore assumed that the use of adjuvant chemotherapy in high-risk pN0 colon cancer patients could result in clinical significant health benefits for individual patients.

The detection of sentinel nodes using a SLNM procedure has been extensively investigated [5-9,11,23-29]. For correct interpretation of these data a gross distinction has to be made between i) *in vivo *and *ex vivo *procedures; ii) immunohistochemical (IHC) or reverse-transcriptase polymerase chain reaction (RT-PCR) occult nodal metastatic disease detection.

The *in vivo *SLNM procedure is characterized by a more difficult technical procedure, most notably during a laparoscopic colonic resection, as a subserosal dye injection is performed preoperatively [23-29]. The accuracy and negative predictive value of *in vivo *SLNM procedures are high and range from 90 to 95% and 93 to 97% respectively [23-29]. Upstaging was observed in 28-35% of H&E pN0 patients [23-29]. The *ex vivo *SLNM procedure is technically much easier, as the procedure is executed extracorporally by injection peritumoral blue dye subserosally or submucosally after which gentle massage of the injection site is performed [6-11]. The *ex vivo *SLNM procedure is characterized also by a high accuracy of 90-100%, and negative predictive value of 80-100%[6-11]. A rate of 19-57% upstaging is observed [6-11].

Although most studies combine data of colon and rectal cancer, a clear distinction in sentinel lymph node mapping between both cancers has been observed in some well-designed studies [6,11,26]. The accuracy of negative predictive value of SLNM in rectal cancer is estimated to be as low as 76% and 65% respectively, and rectal cancers are therefore excluded from inclusion [6].

The method of examination, i.e. IHC or RT-PCR, is also an important determinant of outcome [3,4]. Cytokeratin is a cellular marker for epithelial origin, adhering to constituents of the cytoskeleton of both normal and malignant cells. However, epithelial cells are not normal constituents of lymphoid tissue, and morphological examination of all cytokeratin-positive cells for secondary malignant characteristics is mandatory. Most studies used an IHC methodology, with little similarity in immunohistochemical markers unfortunately [3,4,6-9,24,30]. A high variety in the use of molecular markers for RT-PCR inhibits the selection of the one with highest accuracy and negative predictive value [3,4,23]. The best correlation between micrometastatic disease and worsened DFS and OS has been observed in RT-PCR detected occult disease in comparison to IHC [3,4]. The strength of the evidence of this observed correlation is weak though. Furthermore, RNA is subtracted from halve of a sentinel node in RT-PCR procedures. This unables pathologists the examination of secondary malignant characteristics with H&E colouring. Moreover, the authors of the most recent prospective clinical trial using RT-PCR stated that quantified RT-PCR assays may be of value when cytokeratin immunohistochemistry fails to detect sentinel lymph node metastasis [23]. Therefore, no definitive preference for IHC or RT-PCR methods can be given. For practical arguments, including the possibility for all Dutch hospitals to participate in the study, a controlled clinical trial using an *ex vivo *SLNM procedure with focussed nodal examination by cytokeratin IHC techniques is proposed.

Adjuvant chemotherapy is provided according to the CAPOX (XELOX) protocol (capecitabine (Xeloda^®^) and oxaliplatin(Eloxatin^®^)). This CAPOX (XELOX) protocol is assumed to be comparable in therapeutical effect and toxicity to the currently used FOLFOX-4 protocol, and more patient friendly because of the oral supplementation of chemotherapy (capecitabine) [2].

As primary endpoint is 3-year DFS chosen as surrogate endpoint for overall survival. Colon cancer recurrence is most notably observed in the first years after initial treatment [2,31]. Furthermore, 3-year DFS is recognized as an appropriate surrogate end point for overall 5-year survival in colon cancer trials [32-34].

## Conclusion

Around 30% of all pN0 colon cancer patients will develop locoregional and/or distant disease recurrence. Adjuvant systemic chemotherapy is thought to decrease disease recurrence in high risk pN0_micro+ _patients. A large patient group is subject to this high disease recurrence rate. Therefore, it is justified to perform the proposed trial in a multicenter design in the Netherlands to answer the clinical relevant question of the role of micrometastatic disease on prognosis in stage I-II colon cancer patients.

## Abbreviations

CC: colon cancer; ITC: isolated tumor cell; MM: micrometastasis; SLNM: sentinel lymph node mapping; IHC: immunohistochemistry; RT-PCT: reverse transcriptase-polymerase chain reaction; DFS: disease-free survival; OS: overall survival; pN0: no lymph node metastasis on pathological examination; pN0_micro+/-_: present/absent micrometastasis on pathological examination; pN+: lymph node metastasis on pathological examination; WHO: World Health Organisation; ASA: American Society of Anesthesiologists; NYHA: New York Heart Association; ITT: intention-to-treat; ERAS: enhanced recovery after surgery; CRF: case report form; H&E: hematoxylin and eosin; CAPOX/XELOX: capecitabine(Xeloda^®^) and oxaliplatin; FOLFOX: fluorouracil and oxaliplatin.

## Competing interests

The EnRoute*⊕ *Study is supported through an Educational Grant by Roche Pharmaceuticals.

The authors declare that they have no other competing interests.

## Authors' contributions

DJL drafted the manuscript. KB, CJHV, GJL edited the manuscript. All authors participated in the design of the study. HP performed the statistical analysis. All authors read and approved the final manuscript.

## Authors' information

Daniel J. Lips^1^, Boukje Koebrugge^1^, Gerrit Jan Liefers^2^, Johannes C. van der Linden^3^, Vincent T.H.B.M. Smit^4^, Hans F.M. Pruijt^5^, Hein Putter^6^, Cornelis J.H. van de Velde^2^, Koop Bosscha^1^.

^1 ^Department of Surgery, Jeroen Bosch Hospital, 's-Hertogenbosch, the Netherlands

^2 ^Department of Surgery, Leiden University Medical Center, Leiden, the Netherlands

^3 ^Department of Pathology, Jeroen Bosch Hospital, 's-Hertogenbosch, the Netherlands

^4 ^Department of Pathology, Leiden University Medical Center, Leiden, the Netherlands

^5 ^Department of Medical Oncology, Jeroen Bosch Hospital, 's-Hertogenbosch, the Netherlands

^6 ^Department of Medical Statistics, Leiden University Medical Center, Leiden, the Netherlands

## Pre-publication history

The pre-publication history for this paper can be accessed here:

http://www.biomedcentral.com/1471-2482/11/11/prepub
